# CASK and CaMKII function in the mushroom body α′/β′ neurons during *Drosophila* memory formation

**DOI:** 10.3389/fncir.2013.00052

**Published:** 2013-03-27

**Authors:** Bilal R. Malik, John Michael Gillespie, James J. L. Hodge

**Affiliations:** School of Physiology and Pharmacology, University of BristolBristol, UK

**Keywords:** CASK, CaMKII, memory, *Drosophila*, mushroom body, calcium imaging, autophosphorylation, disease model

## Abstract

Ca^2+^/CaM serine/threonine kinase II (CaMKII) is a central molecule in mechanisms of synaptic plasticity and memory. A vital feature of CaMKII in plasticity is its ability to switch to a calcium (Ca^2+^) independent constitutively active state after autophosphorylation at threonine 287 (T287). A second pair of sites, T306 T307 in the calmodulin (CaM) binding region once autophosphorylated, prevent subsequent CaM binding and inactivates the kinase during synaptic plasticity and memory. Recently a synaptic molecule called Ca^2+^/CaM-dependent serine protein kinase (CASK) has been shown to control both sets of CaMKII autophosphorylation events and hence is well poised to be a key regulator of memory. We show deletion of full length CASK or just its CaMK-like and L27 domains disrupts middle-term memory (MTM) and long-term memory (LTM), with CASK function in the α′/β′ subset of mushroom body neurons being required for memory. Likewise directly changing the levels of CaMKII autophosphorylation in these neurons removed MTM and LTM. The requirement of CASK and CaMKII autophosphorylation was not developmental as their manipulation just in the adult α′/β′ neurons was sufficient to remove memory. Overexpression of CASK or CaMKII in the α′/β′ neurons also occluded MTM and LTM. Overexpression of either *Drosophila* or human CASK in the α′/β′ neurons of the CASK mutant completely rescued memory, confirming that CASK signaling in α′/β′ neurons is necessary and sufficient for *Drosophila* memory formation and that the neuronal function of CASK is conserved between *Drosophila* and human. At the cellular level CaMKII overexpression in the α′/β′ neurons increased activity dependent Ca^2+^ responses while reduction of CaMKII decreased it. Likewise reducing CASK or directly expressing a phosphomimetic CaMKII T287D transgene in the α′/β′ similarly decreased Ca^2+^ signaling. Our results are consistent with CASK regulating CaMKII autophosphorylation in a pathway required for memory formation that involves activity dependent changes in Ca^2+^ signaling in the α′/β′ neurons.

## Introduction

**Figure 1:**
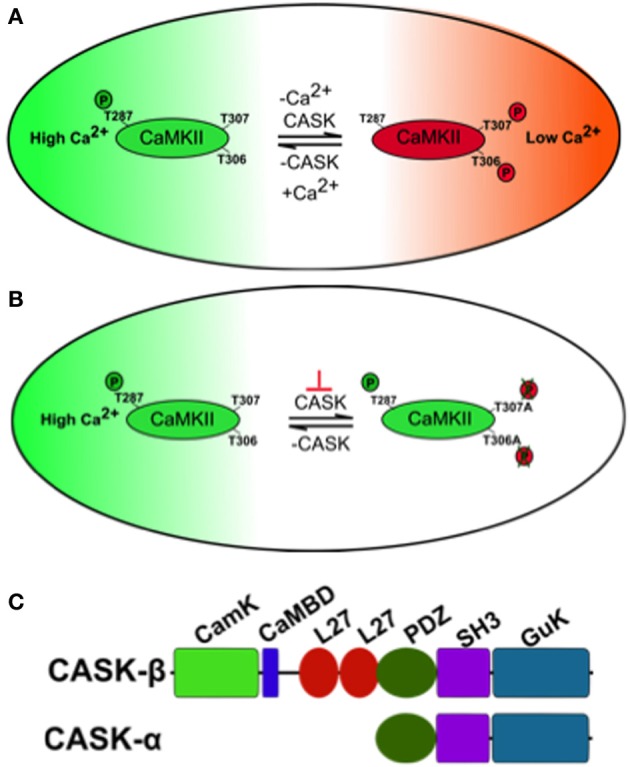
**A model of how CASK regulates CaMKII autophosphorylation during memory. (A)** The large oblong represents a hypothetical neuron shifting between a state of high synaptic activity (high [Ca^2+^], in green) and low synaptic activity (low [Ca^2+^], in red) on the right. The small oblong within the neuron represents a single subunit of the CaMKII dodecamer holoenzyme. Under conditions of high Ca^2+^, Ca^2+^/Calmodulin (CaM) binds CaMKII via the CaM binding site that contains the inhibitory T306 T307 sites hence blocking them from autophosphorylation. This also promotes T287 autophosphorylation (pT287) and the switch to persistently high kinase activity after Ca^2+^ levels fall. Under conditions of low synaptic activity and hence low [Ca^2+^], there is low probability of CaM binding to CaMKII allowing CASK to promote autophosphorylation of the inhibitory T306 T307 (pT306 pT307) sites. This renders the kinase inactive and even if there is a subsequent increase in Ca^2+^/CaM, CaM binding is blocked by pT306 pT307 in the CaM site. Eventually phosphatases will act to remove phosphorylation events and return endogenous CaMKII to its basal state. Therefore, in the absence of CASK there is a decrease in inhibitory pT306 pT307 and an increase in pT287 constitutively active CaMKII, conversely increased CASK promotes inhibitory pT306 pT307 decreasing pT287 and endogenous CaMKII activity. **(B)** Neurons expressing transgenic CaMKII with inhibitory phosphorylation sites mutated to blocking residues (T306A T307A) or with too little CASK due to mutation (depicted by the orange ⊥) result in a form of CaMKII that is unable to switch off. This causes abnormally high transgenic CaMKII activity that subsequently interferes with the physiology of the neuron disrupting memory. **(C)** Predicted domain structure of CASK isoforms, the short isoform CASK-α contains PDZ, SH3, and GUK domains while the long isoform CASK-β contains additional CaMK-like (CamK), Calmodulin binding domain (CaMBD) and L27 domains at its N-terminus. The *CASK-β* null contains a N-terminal deletion that removes a large portion of the 5′UTR and the complete first coding exon including translational start site for *CASK-β* but leaves the downstream promoter and whole of *CASK-α* intact (Slawson et al., 2011). The *uas-CASK* line (Lu et al., 2003; Hodge et al., 2006; Slawson et al., 2011) used in this study expresses the full-length long isoform of CASK (*CASK-β*).

Changes in neural activity and Ca^2+^ signaling in neural circuits of memory centers encode information during memory formation. One molecule critical for these processes is Ca^2+^/CaM serine/threonine kinase II (CaMKII) whose activity is acutely sensitive to changes in Ca^2+^ during long-term potentiation (LTP) underlying hippocampal memory formation (Lisman et al., 2002). Further features that endow CaMKII with its central role in memory formation are its abundance in structures known to be required for memory. For instance, CaMKII is the main protein in the hippocampal post-synaptic density (PSD) (Kelly et al., 1984) and is similarly enriched in the mushroom body memory center of *Drosophila* (Takamatsu et al., 2003; Hodge et al., 2006). Finally CaMKII has also been dubbed “the molecular memory switch”; because after it associates with Ca^2+^/CaM it undergoes a conformational change exposing a T286 on mammalian CaMKII and T287 on *Drosophila* CaMKII that can be autophosphorylated (Figure 1A), resulting in a Ca^2+^ independent constitutively active kinase (Lisman and Zhabotinsky, 2001). Pharmacological blockade or knockout of CaMKII results in mice with deficits in LTP and memory (Silva et al., 1992a,b). Mice expressing Ca^2+^ dependent CaMKII-T286A have no LTP and memory and those expressing CaMKII-T286D also have abnormal LTP and memory (Mayford et al., 1996; Yasuda and Mayford, 2006). A second pair of autophosphorylation events within the CaM binding domain (TT305/6 equivalent to *Drosophila* TT306/7, Figure 1B) occur when Ca^2+^/Calmodulin (CaM) dissociates from CaMKII and are inhibitory as autophosphorylation prevents subsequent CaM binding and hence inhibits CaMKII function. Mice with blocked inhibitory sites (CaMKII-TT305/6AA) show enhanced LTP while CaMKII-TT305/6DD expression also disrupts LTP and memory (Elgersma et al., 2002). In *Drosophila*, there is no *CaMKII* null, which would be expected to be lethal (Park et al., 2002; Mehren and Griffith, 2004), however peptide inhibition of CaMKII led to synaptic defects and memory deficits in the courtship-conditioning assay (Griffith et al., 1993, 1994). Therefore, the control of CaMKII and its autophosphorylation is critical for synaptic plasticity and memory in *Drosophila* and mammals. But the mechanism of regulation of CaMKII autophosphorylation during memory formation is still unclear.

One molecule that in addition to CaM regulates CaMKII autophosphorylation is CASK (Ca^2+^/CaM-dependent serine protein kinase, Figure 1C), a membrane-associated guanylate kinase (MAGUK) scaffolding protein that contains a CaMK-like and Lin-2/Lin-7 (L27) domain in addition to the canonical PDZ [Post-synaptic density protein (PSD95), *Drosophila* disc large tumor suppressor (Dlg1), and Zonula occludens-1 protein (Zo-1)], SH3 (SRC Homology 3), and GUK (guanylate kinase) domains with the CaMK and GUK domains likely kinase dead in *Drosophila* (Hata et al., 1996; Lu et al., 2003). The CaMK domain of CASK has low levels of Ca^2+^/CaM independent activity against neurexin that unlike other kinases is magnesium independent (Mukherjee et al., 2008). Again the GUK domain of mammalian CASK encodes a pseudokinase. Two isoforms of CASK are present in flies, a long form, *CASK-β* and a short isoform, *CASK-α* (Figure 1C). The long form *CASK-β* contains the additional N-terminal CaMK-like and L27 domains, while the short form *CASK-α* contains just the canonical PDZ, SH3, and GUK domains which are common to both isoforms, and shows homology to the vertebrate MPP protein (Slawson et al., 2011). CASK-β associates with CaMKII at synapses and in the absence of Ca^2+^/CaM promotes TT306/7 phosphorylation (Figure 1A), inactivating the kinase (Lu et al., 2003). Deletion of CASK in mice results in lethality, preventing their use in modeling CASK function in synaptic plasticity and memory (Atasoy et al., 2007). Flies completely lacking CASK are viable, have decreased levels of synaptic CaMKII-TT306/7 autophosphorylation and display abnormal habituation (Lu et al., 2003). Furthermore, CASK mutants increase T287 autophosphorylation thereby endowing CASK with the ability to regulate the CaMKII switch to Ca^2+^ independence (Hodge et al., 2006). CASK is expressed throughout the fly brain including the mushroom bodies (Martin and Ollo, 1996; Lu et al., 2003). In this study we determine the role of CASK and CaMKII autophosphorylation in memory and measure the accompanying changes in mushroom body Ca^2+^ signaling.

## Materials and methods

### Drosophila stocks

Flies were grown on cornmeal molasses agar medium under standard conditions. *CASK-β null*, *uas-CASK (10,20MI), uas-CaMKII, uas-CaMKII-T287D, uas-CaMKII-T287A, uas-CaMKII-TT306/7AA, Df(3R)x307*, and *Df(3R)x313* (Lu et al., 2003; Slawson et al., 2011) were kind gifts from Dr. Leslie Griffith (Brandeis University, US). *uas-CASK-RNAi* flies (stock #104793) were obtained from the Vienna *Drosophila* Stock Center (VDRC). *OK107-Gal4, c305a-Gal4, MB247-Gal4*, and wildtype flies [*CantonSw*-, (*CSw*-)] were from Dr. Scott Waddell (Oxford University, UK). All *CASK* and *CaMKII* mutants, *Gal4*, and *UAS* lines were outcrossed with the *CSw*- line for at least six generations prior to behavioral experiments. *GCaMP3.1* flies were a gift from Dr. Loren Looger (Janelia farm, VA, US). *MB247-Gal4; tubulin-Gal80*^*ts*^ and *OK107-Gal4; tubulin-Gal80*^*ts*^ were obtained from Dr. Yi Zhong (Cold Spring Harbor Laboratories, US). *uas-CaMKII-RNAi* and *CaMKII-Gal4* flies were obtained from Dr. Sam Kunes (Harvard University, US).

### Cloning

Human *CASK* cDNA isolated from human cerebellum was obtained from imaGenes (IMAGE full-length cDNA clone IRCMp5012G0614D, http://www.imagenes-bio.de) in a *pCR4-TOPO* vector. Forward (5′-*CACC ATG GCC GAC GACGAC*-3′) and reverse (5′-*CTA ATA GAC CCA GGA GAC AGG*-3′) primers (0.4 μL at 0.5 μM, Invitrogen), *dNTPs* (200 μM), and *CASK* cDNA (1 μl) was added to ddH_2_O (13.4 μl) before addition of High fidelity Phusion DNA polymerase (0.2 μl, Finnzymes). The following reaction conditions were then used for PCR: 98°C for 30 s, 98°C for 10 s, 61°C for 20 s, 72°C for 60 s (25 cycles), 72°C for 5 min. The resultant PCR product was used for *pENTR™* directional TOPO® cloning (Invitrogen) to create the plasmid *pEntr-CASK*. This was used to transfect α-Select Gold *E. Coli* (Bioline). This plasmid was then sequenced (Geneservice, London, http://www.geneservice.co.uk) and used in the Gateway LR cloning reaction (Invitrogen) with a *pTW* plasmid. The plasmid *pTW-CASK* was used for germline transformation (Bestgene, US) by microinjection into *Drosophila* embryos.

### Behavior experiments

Behavior experiments were carried out at 25°C, 70% relative humidity and under dim red light. For *Gal80*^*ts*^ (TARGET) experiments the flies were grown at 18°C that allowed *Gal80*^*ts*^ inhibition of *Gal4*. Adult flies were collected everyday in the evening and maintained for another three days at 30°C. These flies were trained and tested at 30°C that relieved *Gal80*^*ts*^ inhibition allowing the expression of transgenes (McGuire et al., 2003). To measure learning (2 min memory) a mixed population of about one hundred 2–3 days (4 days for TARGET experiments) day old flies received one cycle of training during which they were exposed sequentially to one odor [conditioned stimulus, CS+; 3-octanol (1:100) or 4-methyl-cyclohexanol, 1:67] paired with electric shock (60V DC) (unconditioned stimulus, US) and then to a second odor (CS-odor) without electric shock. The flies were then allowed to choose between the two odors for 120 s in the T-maze (Tully and Quinn, 1985). To measure middle-term memory (MTM) flies were given one cycle of training and then stored in food containing vials for 3 h before they were tested as in learning experiments. A performance index (PI) was calculated as the number of flies avoiding the CS+ minus number of flies avoiding the CS-, divided by the total number of flies that participated in the test. A score of 1.0 would be equivalent to 100% learning, where all the flies avoided the CS+. In contrast a 50:50 distribution would give a PI of zero (no learning). For long-term memory a custom built maze was used which allowed simultaneous training of several batches of flies. The flies were administered five cycles of training either with an inter-cycle interval of 15 min (spaced) or without any inter-cycle interval (massed). They were then kept at 18°C until tested. Prior to testing, the flies were moved to 25°C and allowed to acclimatize for at least 1 h. For long-term memory (LTM), memory was assessed 24 h after training. All statistical analysis for behavioral data was performed and plotted with Graphpad Prism (Graphpad software, Inc) software.

### Calcium imaging

Ca^2+^ imaging on dissected adult brains was performed as described previously (Ruta et al., 2010; Tessier and Broadie, 2011). Briefly, the fly brains were dissected in HL3.1, tethered to the bottom of a petri dish containing 5 ml of HL3.1. Images were collected using an Axio Examiner Z1 microscope (Zeiss) using a 10× water immersion objective and Axiovision software. The brains were stimulated by gently adding 500 μl of 65 mM KCl in HL3.1 to the dish while the images were captured at 340 msec/frame.

### Image analysis

Image analysis was performed using the single channel ratio analysis of the physiology module of AxioVs40 V 4.8.0.0 (Zeiss). Regions of interest were selected by drawing around the mushroom bodies α′/β′ neurons and the fluorescence values were obtained. An initial reference fluorescence (*F*_*o*_) value of was calculated by averaging the fluorescence of first ten frames. Percent change in fluorescence, %Δ*F*/*F*, was calculated for each time point, which is given by [(*F*-*F*_*o*_/*F*_*o*_) × 100], where *F* is fluorescence at a given time. A ratio table was generated and the values were plotted as a function of number of time.

### Western blotting and *RNAi* validation

Extracts were prepared by freezing ten fly heads from either wildtype or *Elav-Gal4 > uas-RNAi* in liquid nitrogen followed by homogenization in 50 μl of lysis buffer (50 mM Tris, pH 7.4, 150 mM NaCl, 1% Triton-x-100, 5 mM EDTA, 0.1% SDS, 1 mM Na_2_VO_3_, and complete mini protease inhibitor (Amersham Biosciences). The homogenate was incubated on ice for 10 min and then centrifuged at 14000 rpm. Supernatant was collected and mixed with 50 μl sample buffer. 15 μl of this sample were loaded per well. Following transfer to a nitrocellulose membrane, the membrane was probed with rabbit anti-CASK 1:800 antibodies. Bands were visualized using horseradish peroxidase-conjugated secondary antibodies (Amersham Biosciences) and enhanced chemiluminescence reagents (ECL, Amersham Biosciences). In order to validate the *RNAi* constructs, the CASK sequence from VDRC and the CaMKII sequence (Ashraf et al., 2006) were used in BLAST searches of the NCBI database and only the appropriate gene of interest came up as a significant hit suggesting no off-targets.

### Immunohistochemistry

Immunohistochemistry was performed essentially according to previously published protocols (Hodge et al., 2006). Briefly, the fly adult brains were dissected for 4–8 days old flies in HL3.1. The isolated brains were then fixed in 4% paraformaldehyde for 1 h followed by two washes with HL3.1-Tx (HL3.1 containing 0.1% Triton-X-100) for a total of 1 h. The brains were then blocked for 1 h with 0.1% bovine serum albumen (BSA) and 0.1% normal goat serum (NGS) in HL3-Tx. Brains were incubated overnight at 4°C with 1:40 dilution of a rabbit anti-CASK antibody (Lu et al., 2003) or 1:100 mouse anti-CaMKII (Takamatsu et al., 2003). Following an overnight washing HL3.1-Tx at 4°C the brains were then incubated with 1:400 anti-rabbit Alexa-648 or with 1:400 goat anti-mouse Alexa-488 (Invitrogen) secondary antibodies overnight. Following an overnight HL3.1-Tx wash the brains were mounted in Vectashield (Vector laboratories) and stored at 4°C in the dark until they were imaged using a Leica TCS SP5 confocal microscope (Wolfson Bioimaging facility, University of Bristol). The images were then examined using Velocity imaging software (PerkinElmer) and projections were generated using the image processing software ImageJ (NIH).

### Sensorimotor controls

The odor acuity and shock reactivity were determined for all genotypes used in this study, as described previously (Tully et al., 1994). Briefly, for odor acuity ~80–100 flies were introduced into the T-maze. After 90 s the flies were taken to the choice point where they were allowed 2 min to make a choice between pure odors and air. The flies were then collected and counted. The percent avoidance was calculated by dividing the flies that chose odor by the total number of flies that participated in the test. For shock reactivity, flies were introduced into the shock chamber. After 90 s of rest they were given a 60 V DC electric shock from which time they were allowed to escape to a similar tube without electric shock on the other side. They were given 2 min to make a choice and then collected and counted. The percent shock avoidance was calculated by dividing the number of flies that avoided the shock by escaping the shock tube by the total number of flies in the experiment. The flies that remained in the central chamber were considered to have escaped the electric shock.

## Results

### CASK-β isoform containing CaMKII-like and L27 domains is required for middle-term memory

**Figure 2:**
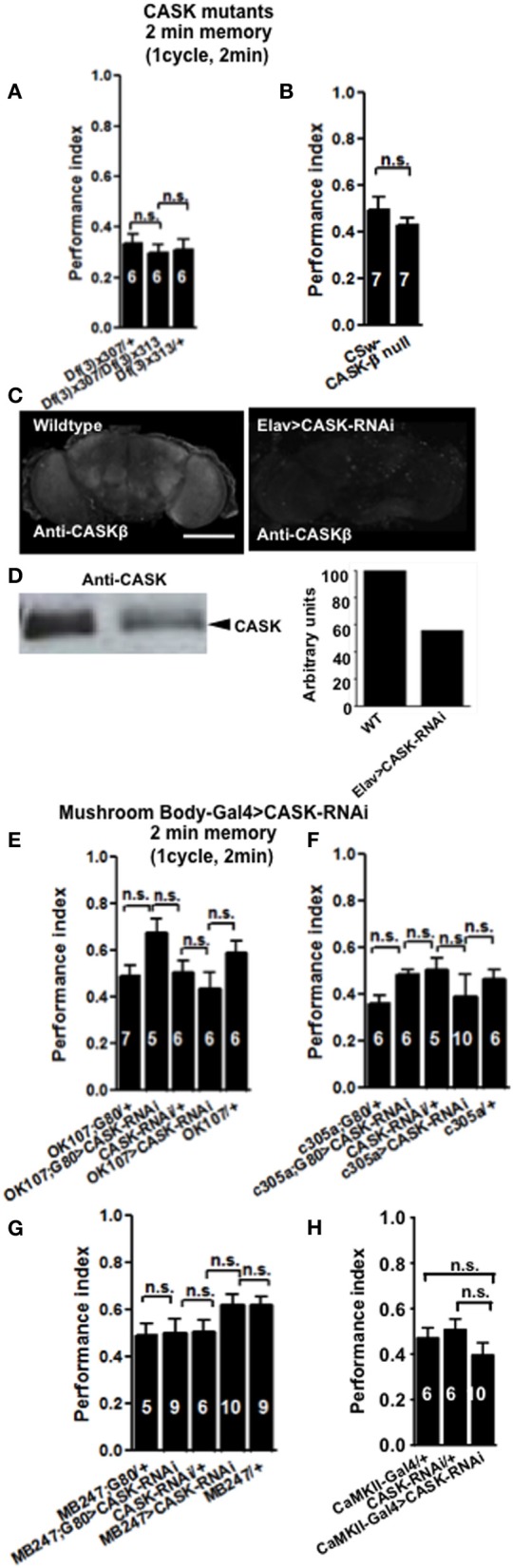
**CASK is not required for learning. (A)** Learning or initial (2 min) short-term memory (STM) was measured immediately after administering one cycle of shock-odor training. Flies lacking all forms of *CASK [Df(3)x307/Df(3)x313]* learned equally well to heterozygote negative controls *[Df(3)x307/+* or *Df(3)x313/+]*. Data were analyzed using One-Way ANOVA followed by a Tukey's *post-hoc* test. In all figures the numbers denote n (typically ~100 flies used for each n), n.s. is not significant (*p* > 0.05). The brackets below the significance label denote the genotypes being compared. **(B)** Flies lacking *CASK-β* (*CASK-β* null), learned equally well to avoid the shock-paired odor as wildtype negative control. **(C)** Compared to wildtype, pan-neuronal (*elav-Gal4*) expression of *uas-CASK-RNAi* leads to reduced CASK-β immunofluorescence in whole mount adult brains. (**D**) Western blot of cell lysates from wildtype or *elav-Gal4*, *uas-CASK-RNAi* heads showed a similar reduction in CASK-β. Quantification of the intensity of the CASK-β band showed a ~50% reduction in CASK-β expression in *elav-Gal4*, *uas-CASK-RNAi*. **(E)** Flies with targeted reduction of *CASK* throughout their mushroom body (*OK107-Gal4 > uas-CASK-RNAi*) or just in the adult mushroom body (*OK107-Gal4; Gal80*^*ts*^ > *uas-CASK-RNAi*) displayed learning comparable to heterozygous wildtype negative controls. **(F)** Flies with targeted reduction of *CASK* in the mushroom body α′/β′ neurons (*c305-Gal4 > uas-CASK-RNAi*) or just in the adult mushroom body α′/β′ neurons (*c305a-Gal4; Gal80*^*ts*^ > *uas-CASK-RNAi*) displayed learning comparable to heterozygous wildtype negative controls. **(G)** Flies with targeted reduction of *CASK* in the mushroom body α/β and γ neurons (*MB247-Gal4 > uas-CASK-RNAi*) or just in the adult mushroom body α/β and γ neurons (*MB247-Gal4; Gal80*^*ts*^ > *uas-CASK-RNAi*) displayed learning comparable to heterozygous wildtype negative controls. **(H)** Flies with targeted reduction of *CASK* in *CaMKII* neurons (*CaMKII-Gal4 > uas-CASK-RNAi*) displayed learning comparable to heterozygous wildtype negative controls. All data were analyzed using One-Way ANOVA followed by a Tukey's *post-hoc* test.

In order to see if CASK plays a role in learning and memory flies were tested using the olfactory aversive conditioning assay (Tully and Quinn, 1985). We used deficiency lines: *Df(3R)x307* and *Df(3R)x313* which contain large chromosomal deficiencies both lacking *CASK* [called *camguk (cmg)* or *caki*]. A cross between the two lines generates transheterozygote flies with only a short fragment of chromosome deleted that includes the whole of the *CASK* locus, therefore null for both *CASK-α* and *CASK-β* (Martin and Ollo, 1996). All *CASK* mutant genotypes learned to avoid the shock-paired odor similar to controls when tested 2 min after training (Figure 2A). In order to investigate the role of the different *CASK* isoforms in learning (2 min memory) we used a mutant (*CASK-β* null) that completely removes the long isoform of *CASK* (*CASK-β*) but leaves the short (*CASK-α*) isoform intact (Figure 1C; Slawson et al., 2011). These mutant flies also did not show any defects in 2 min memory tested after 2 min of administering one training cycle (Figure 2B).

The majority of the *CASK* and *CaMKII* mutant genotypes tested showed normal shock reactivity and olfactory acuity demonstrating that any performance deficit was due to a defect in signal processing required for memory as opposed to a peripheral defect preventing the fly from being able to perform the behavioral task (Table 1). *CASK-β* null, *Df(3)x313/Df(3)x307* and the *CASK* heterozygous control deficiency line *Df(3)x313/+* reacted abnormally to electric shock. However, all the *CASK* and *CaMKII* mutant genotypes showed normal learning confirming that these flies are healthy and have mushroom bodies that are capable of detecting odor, respond to shock normally and able to support initial learning, therefore the data shown in Figures 2A,B are negative controls for this issue. In addition none of the flies displayed any obvious developmental defect and neither displayed a wing phenotype or sluggishness (Park et al., 2002). This was reflected in the fact they were wildtype for peripheral controls (Table 1) and learning (Figures 2, 4), so therefore were able to choose to move away from the shock-paired odor in the T-maze the same as wildtype flies (Figures 2, 4).

**Table 1:** **The sensorimotor controls for *CASK* and *CaMKII* transgenic flies**.

	**Odor avoidance**		**Percent shock avoidance**
	**MCH**	**OCT**	
	**Mean ± *SEM***	**Mean ± *SEM***	**Mean ± *SEM***
WT Control	0.77 ± 0.06	0.69 ± 0.03	62.9 ± 3.8
MB247/+	0.6 ± 0.08	0.59 ± 0.03	62.7 ± 3.5
c305a/+	0.59 ± 0.03	0.54 ± 0.04	93.9 ± 2.1
OK107/+	0.65 ± 0.05	0.58 ± 0.07	87 ± 2
CASK-RNAi/+	0.59 ± 0.07	0.68 ± 0.1	85.7 ± 1.7
MB247 > CASK-RNAi	0.58 ± 0.05	0.84 ± 0.07	73.4 ± 3.7
c305a > CASK-RNAi	0.61 ± 0.03	0.83 ± 0.11	79.4 ± 3.2
OK107 > CASK-RNAi	0.63 ± 0.06	0.87 ± 0.04	79 ± 2.4
MB247;G80 >	0.64 ± 0.04	0.61 ± 0.05	75.7 ± 4.1
CASK-RNAi			
OK107;G80 >	0.71 ± 0.02	0.65 ± 0.05	78.5 ± 3.5
CASK-RNAi			
Df(3)x307/+	0.65 ± 0.03	0.53 ± 0.1	72 ± 5.6
Df(3)x313/+	0.6 ± 0.05	0.52 ± 0.07	41.5 ± 1.4^*^
Df(3)x307/Df(3)x313	0.63 ± 0.08	0.72 ± 0.05	43.5 ± 8.9^*^
T287D/+	0.61 ± 0.07	0.61 ± 0.05	70 ± 6.1
MB247 > T287D	0.59 ± 0.05	0.64 ± 0.16	64.9 ± 5.4
c305a > T287D	0.65 ± 0.13	0.66 ± 0.04	61.5 ± 11.7
OK107 > T287D	0.87 ± 0.02	0.73 ± 0.03	69 ± 2.6
MB247;G80 > T287D	0.7 ± 0.03	0.61 ± 0.11	75.5 ± 7.6
OK107;G80 > T287D	0.7 ± 0.07	0.68 ± 0.08	81 ± 3.1
CaMKII-Gal4 > T287D	0.48 ± 0.04	0.51 ± 0.12	93 ± 3.5
CASK-β null	0.61 ± 0.07	0.53 ± 0.08	35.9 ± 3.6^*^
CASK;CASK-β null	0.53 ± 0.05	0.52 ± 0.09	37 ± 6.9^*^
c305a;CASK-β null	0.49 ± 0.09	0.49 ± 0.04	72.5 ± 1.7
c305a >	0.63 ± 0.06	0.65 ± 0.07	60.3 ± 4.4
CASK;CASK-β null			
CASK/+	0.64 ± 0.1	0.56 ± 0.05	73.1 ± 3.6
MB247 > CASK	0.57 ± 0.06	0.64 ± 0.12	93.2 ± 2.3
c305a > CASK	0.86 ± 0.03	0.59 ± 0.05	71.9 ± 3.2
OK107 > CASK	0.86 ± 0.03	0.59 ± 0.05	82.2 ± 2.2
T306A T307A/+	0.56 ± 0.03	0.55 ± 0.1	92.5 ± 1
MB247 > T306A T307A	0.55 ± 0.07	0.64 ± 0.05	89.8 ± 3.7
c305a > T306A T307A	0.56 ± 0.06	0.6 ± 0.02	91.8 ± 1.7
OK107 > T306A T307A	0.62 ± 0.03	0.57 ± 0.03	88.8 ± 1.4
CaMKII/+	0.64 ± 0.05	0.6 ± 0.11	79.6 ± 2.4
MB247 > CaMKII	0.58 ± 0.02	0.52 ± 0.08	87 ± 3.8
c305a > CaMKII	0.67 ± 0.14	0.52 ± 0.04	82.7 ± 2.8
OK107 > CaMKII	0.74 ± 0.08	0.5 ± 0.03	92.9 ± 1.2
CaMKII-Gal4 > CaMKII	0.48 ± 0.03	0.51 ± 0.11	93 ± 3.5
T287A/+	0.43 ± 0.04	0.61 ± 0.08	85.5 ± 2.2
MB247 > T287A	0.59 ± 0.14	0.41 ± 0.08	72.5 ± 7.5
c305a > T287A	0.51 ± 0.12	0.53 ± 0.07	66.8 ± 1.8
OK107 > T287A	0.59 ± 0.14	0.6 ± 0.05	69.5 ± 7.6
CaMKII-RNAi/+	0.62 ± 0.12	0.6 ± 0.06	90.5 ± 0.5
MB247 > CaMKII-RNAi	0.57 ± 0.08	0.55 ± 0.12	95.8 ± 2.4
c305a > CaMKII-RNAi	0.62 ± 0.07	0.55 ± 0.12	92.3 ± 0.3
OK107 > CaMKII-RNAi	0.63 ± 0.12	0.54 ± 0.12	89 ± 4

CASK and CaMKII transgenic flies showed normal odor acuity for 3-Octanol (OCT) and methyl-cyclohexanol (MCH). However, some of the CASK genotypes have reduced shock reactivity compared to wildtype negative control (

* p < 0.05).

Finally, we decided to investigate the effect of mushroom body specific reduction of *CASK* on learning. *Drosophila* mushroom bodies consist of three different classes of intrinsic neurons (α/β, α′/β′, and γ) that extend their axons into the five lobes of neuropil (Davis, 2011). We used a *CASK-RNAi* line which reduces the expression of CASK by ~50% (Figures 2C,D) to test if reduction of CASK in the mushroom body has an effect on learning in flies. Expression of *CASK-RNAi* transgene in either all mushroom body neurons [*OK107-Gal4* (Connolly et al., 1996)], mushroom body α′/β′ neurons [*c305a-Gal4* (Krashes et al., 2007)], mushroom body α/β and γ neurons [*MB247-Gal4* (Zars et al., 2000)], or using a *CaMKII-Gal4* [that expresses in the mushroom body α/β, α′/β′, and dorsal anterior lateral (DAL) neurons, (Chen et al., 2012)] drivers did not lead to a significant decrease in 2 min memory (Figures 2E–H).

**Figure 3:**
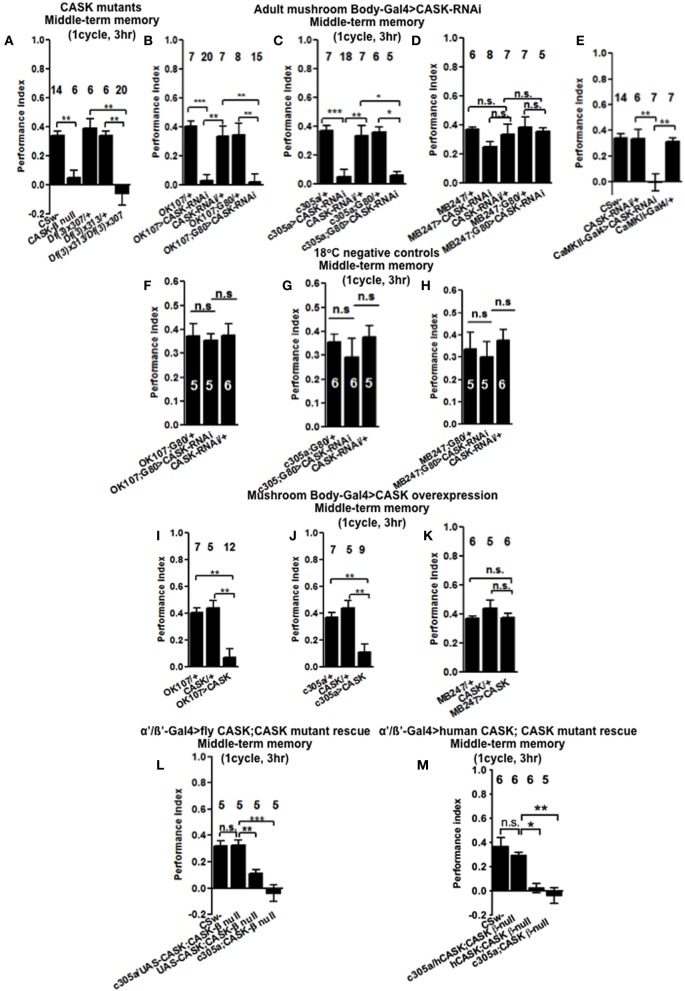
**CASK functions in the mushroom body α′/β′ neurons during middle-term memory formation. (A)** MTM measured 3 h post-training was completely removed in *CASK-β* null flies. Similarly transheterozygous [*Df(3)x313*/*Df(3)x307*] flies that lack both *α-CASK* and *β-CASK* have a similar reduction in MTM compared to wildtype or heterozygote negative controls [*Df(3)x313*/+ or *Df(3)x307/+*]. **(B)** Flies with *uas-CASK-RNAi* expressed throughout their mushroom body using *OK107-Gal4* show a reduction in MTM compared to heterozygous wildtype negative controls. Adult specific reduction in mushroom body *CASK* using *OK107-Gal4; Gal80*^*ts*^ was sufficient to reduce MTM. **(C)** Flies expressing *uas-CASK-RNAi* in their α′/β′ mushroom body neurons (*c305a-Gal4*) show a reduction in MTM. Reduction of CASK just in the adult α′/β′ neurons using *Gal4-c305a; Gal80*^*ts*^ was sufficient to cause the reduction in MTM. **(D)** Reduction of CASK in α/β and γ neurons using *MB247-Gal4* did not affect MTM. Adult specific reduction of CASK in α/β and γ neurons using *MB247-Gal4; Gal80*^*ts*^ also did not affect MTM. **(E)** *CASK-RNAi* expression using *CaMKII-Gal4* also lead to a MTM defect compared to wildtype. **(F)** Flies that contained *Gal80*^*ts*^ in combination with either *Gal4-OK107* **(G)** *c305a-Gal4* **(H)** *MB247-Gal4* were reared and tested at 18°C, a temperature that prevented the expression of the *CASK-RNAi* (hence these are negative control experiments) displayed MTM similar to heterozygous wildtype controls. **(I)** MTM was completely removed in flies overexpressing full-length *CASK* throughout their mushroom body. **(J)** Overexpression of CASK in α′/β′ neurons was sufficient to cause the decrease in MTM. **(K)** α/β and γ neuron overexpression of *CASK* did not affect MTM. **(L)** Expression of *CASK* in mushroom body α′/β′ neurons in a *CASK-β* null background (*c305a-Gal4, uas-CASK; CASK-β* null) rescued the reduction in MTM seen in the *CASK-β* null mutants [*uas-CASK; CASK-β* null and *c305a-Gal4; CASK-β* null (the positive controls) compared to wildtype (the negative control)] to the same level as wildtype. **(M)** Overexpression of human *CASK* in the mushroom bodies α′/β′ neurons in a fly otherwise completely lacking *CASK-β* rescued the reduction in MTM seen in the *CASK-β* null mutants (*uas-CASK; CASK-β* null and *Gal4-c305a; CASK-β* null compared to wildtype) to the same level as wildtype. All data were analyzed using One-Way ANOVA followed by a Tukey's *post-hoc* test. n.s. is not significant, (*p* > 0.05), ^*^p < 0.05, ^**^p < 0.01, and ^***^p < 0.001.

We then tested flies 3 h after one cycle of training (Figure 3A), *CASK-β* null reduced MTM to a similar extent as *Df(3)x307/Df(3)x313*. This showed that deletion of *CASK-β* alone was sufficient to cause the MTM defect, indicating an important role for the CaMK-like and L27 domains of CASK in MTM. Flies with *CASK* knockdown in either all mushroom body neurons (Figure 3B) or just α′/β′ neurons (Figure 3C) similarly showed a drastic reduction in MTM, while restricting expression to the remaining α/β and γ neurons had no effect (Figure 3D). This suggests CASK specifically controls memory formation via the α′/β′ neurons. In order to distinguish the role of CASK in mushroom body development as opposed to an acute physiological role in signaling underlying memory we restricted the reduction of *CASK* to just the adult mushroom body using the TARGET system (McGuire et al., 2003). Reduction of *CASK* specifically in the adult mushroom body was sufficient to cause the reduction in MTM showing that the effects are post-developmental (Figure 3B). Again we confirmed that this deficit in MTM resulted from a function of CASK in the adult α′/β′ neurons (Figure 3C) as opposed to the adult α/β and γ neurons (Figure 3D). The negative control flies reared and tested at 18°C, conditions where there was no transgene expression (Shuai et al., 2010) showed normal MTM (Figures 3F–H).

As previous work has showed that CASK influences plasticity and behavior via regulation of CaMKII autophosphorylation (Lu et al., 2003; Hodge et al., 2006) we used the *CaMKII*-specific promoter that appears to express in the mushroom body α/β, α′/β′, and DAL neurons and has been used to follow the changes in CaMKII transcription occurring during LTM (Chen et al., 2012). Knockdown of *CASK* in these CaMKII neurons was sufficient to completely remove MTM (Figure 3E). We believe it is the α′/β′ neurons of the *CaMKII-Gal4* expression pattern that are most critical for mediating CASK and CaMKII effects on memory, as CASK and CaMKII memory phenotypes map to α′/β′ (*c305a-Gal4*) neurons with α/β (*MB247-Gal4*) neurons having little effect and the DAL neurons thought to only affect certain aspects of LTM (Chen et al., 2012). The data suggests that CASK is needed in a subset of neurons that express CaMKII in order to get memory formation. In order to determine if increased levels of CASK also disrupted MTM we expressed *uas-CASK*, the cDNA corresponding to the long isoform called *CASK-β* (Figure 1C; Lu et al., 2003; Hodge et al., 2006; Slawson et al., 2011) throughout the mushroom body. This resulted in a dramatic reduction in MTM (Figure 3I), which again could be localized to the α′/β′ neurons (Figure 3J) as opposed to the α/β and γ neurons where CASK overexpression had no effect (Figure 3K). Since the effects of *CASK* knockdown were also localized to the mushroom body α′/β′ neurons, we tested whether expressing the *Drosophila CASK* transgene in these neurons in a *CASK-β* null fly, would return their memory to normal (Figure 3L). Compared to *CASK-β* null mutant flies with *c305a-Gal4* alone or *uas-CASK* alone, mushroom body α′/β′ expression of *CASK* in the *CASK-β* null background fully rescued the MTM defect to a level indistinguishable from wildtype, confirming that CASK signaling in mushroom body α′/β′ is necessary and sufficient for *Drosophila* MTM formation.

### Human *CASK* overexpression in mushroom bodies α′/β′ neurons is sufficient to restore the memory of *CASK* null flies to wildtype

As human CASK and CaMKII display a high degree amino acid residue identity to *Drosophila* CASK (74% identical) and CaMKII (79% identical), it is likely that they might function in a similar way in both organisms (Cho et al., 1991; Hsueh, 2006). In order to test this hypothesis we overexpressed human *CASK* in mushroom body α′/β′ neurons of flies that otherwise express no *CASK-β*. Whereas *CASK-β* null flies almost completely lack MTM, overexpression of human *CASK* just in mushroom body α′/β′ neurons was sufficient to return memory to levels indistinguishable to wildtype (Figure 3M). This indicates that *Drosophila* and human CASK show conserved neuronal function in memory formation.

### Levels of CaMKII autophosphorylation regulate middle-term memory formation

**Figure 4:**
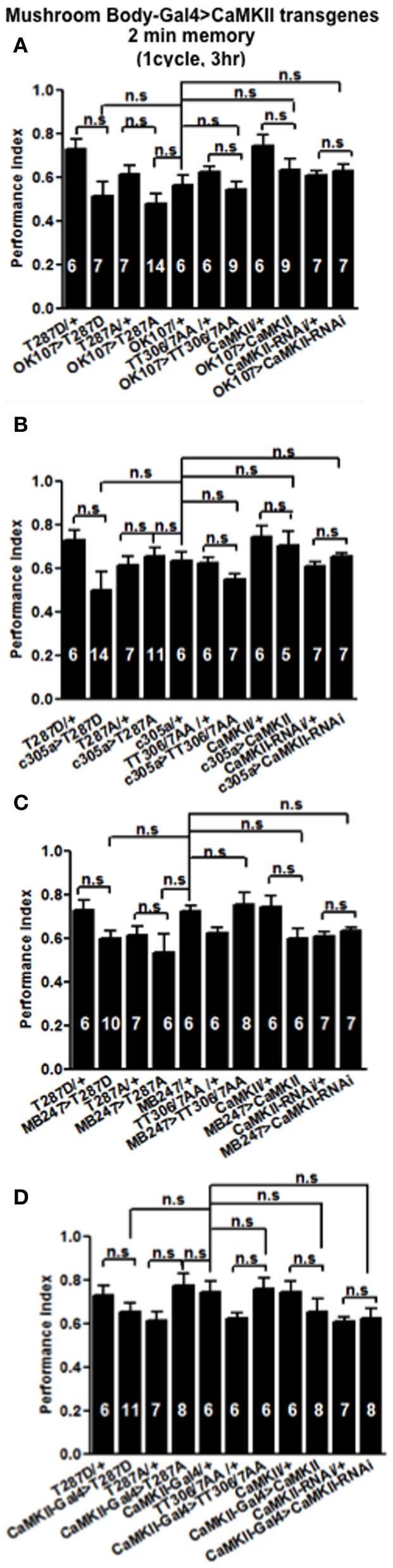
**CaMKII autophosphorylation in the mushroom body is not required for initial memory formation.** Initial memory or learning was measured immediately (2 min) after one cycle training. Flies expressing *CaMKII* transgenes either throughout the mushroom body **(A)**, in the α′/β′ neurons **(B)**, the α/β, and γ neurons **(C)** or CaMKII neurons **(D)** all learned similar to heterozygous wildtype negative control. Data were analyzed using One-Way ANOVA followed by a Tukey's *post-hoc* test.

**Figure 5:**
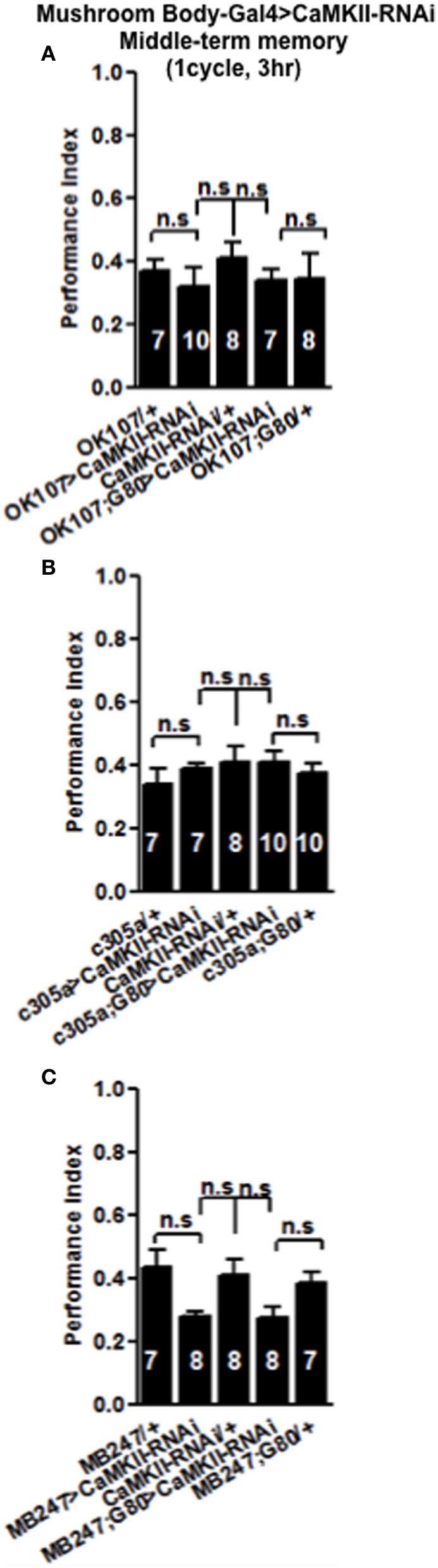
**Levels of CaMKII in the mushroom body are not important for middle-term memory formation.** MTM was equal in flies with reduced *CaMKII* either, throughout the mushroom body **(A)** the α′/β′ neurons **(B)** or the α/β and γ neurons **(C)** compared to heterozygous wildtype negative controls. Data were analyzed using One-Way ANOVA followed by a Tukey's *post-hoc* test.

**Figure 6:**
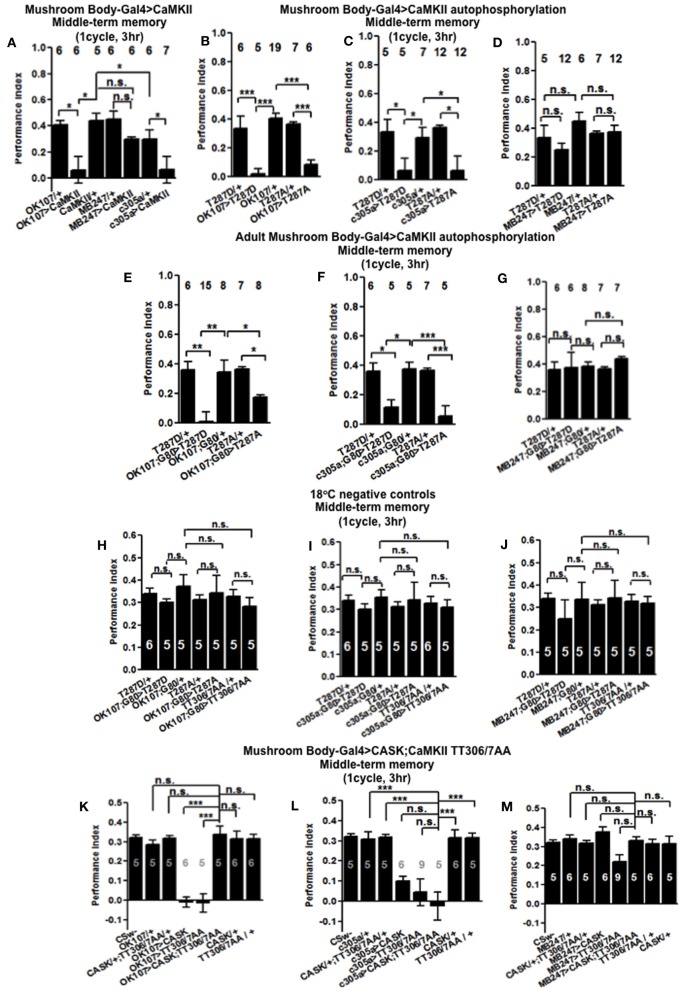
**CaMKII autophosphorylation in the mushroom body α′/β′ neurons are critical for middle-term memory. (A)** Overexpression of *CaMKII* throughout the mushroom body or just in the α′/β′ neurons significantly decreased MTM, while expression in the α/β and γ neurons had little effect. **(B)** Overexpression of constitutively active *CaMKII-T287D* or Ca^2+^ dependent *CaMKII-T287A* throughout the mushroom body significantly decreased MTM. **(C)** Overexpression of *CaMKII-T287D* or *CaMKII-T287A* just in the α′/β′ neurons significantly decreased MTM, **(D)** while expression in the α/β and γ neurons had little effect. **(E)** Adult specific mushroom body expression of *CaMKII-T287D* or *-T287A* with *OK107-Gal4; Gal80*^*ts*^ lead to a reduction in MTM compared to heterozygous wildtype negative controls. **(F)** Adult specific α′/β′ neuron expression of *CaMKII-T287D* or *-T287A* with *c305a-Gal4; Gal80*^*ts*^ was sufficient to cause the reduction in MTM. **(G)** Expression of *CaMKII-T287D* or *-T287A* in the remaining adult α/β and γ neurons with *MB247-Gal4; Gal80*^*ts*^ did not affect MTM. Performance of flies that contained *Gal80*^*ts*^ in combination with *OK107-Gal4* **(H)** *c305a-Gal4* **(I)** and *MB247-Gal4* **(J)** and that were reared and tested at 18°C; a temperature that prevented the expression of the *CaMKII* transgenes and hence is a negative controls had normal MTM. **(K)** Expression of *uas-CaMKII-T306A T307A* or *CASK* alone throughout the mushroom body reduced MTM (e.g., the positive controls) compared to heterozygous wildtype negative controls. Expression of *CaMKII-TT306/7AA* and *CASK* throughout the mushroom body (*OK107-Gal4*) rescued the MTM deficit seen with expression of either transgene alone to wildtype. **(L)** Expression of *uas-CaMKII-T306A T307A* in the α′/β′ neurons was sufficient to reduce MTM compared to controls. However, simultaneous expression of *CASK* and *CaMKII-TT306/7AA* using *c305a-Gal4* was not sufficient to rescue this defect. **(M)** Expression of *uas-CaMKII-T306A T307A* in the α/β and γ neurons did not affect MTM. All data were analyzed using One-Way ANOVA followed by a Tukey's *post-hoc* test. n.s. is not significant, (*p* > 0.05), ^*^p < 0.05, ^**^p < 0.01, and ^***^p < 0.001.

In order to see if the CaMKII levels and autophosphorylation are important for aversive olfactory learning and memory we expressed a range of *CaMKII* transgenes in the mushroom body. These included a transgene that overexpresses *CaMKII* (Koh et al., 1999), a *CaMKII-hairpin* that allows targeted reduction of *CaMKII* (Ashraf et al., 2006; Akalal et al., 2010; Chen et al., 2012), a Ca^2+^-independent constitutively active *CaMKII-T287D*, a Ca^2+^ dependent *CaMKII-T287A* (Park et al., 2002), and *CaMKII-TT306/7AA* containing phospho-blocking mutations of its inhibitory phosphorylation sites (Lu et al., 2003). We found that mushroom body expression of these transgenes did not affect learning with the avoidance of the shock-paired odor being similar between mutant and wildtype flies (Figures 4A–D) similar to what we found for all *CASK* genotypes. In order to see if the level of CaMKII in the mushroom body is important for MTM, we expressed *CaMKII-hairpin* in different parts of the mushroom body, however, none had a significant reduction in MTM compared to the heterozygote wildtype negative control (Figures 5A–C). However, when *CaMKII* is overexpressed throughout the mushroom body, there was a significant reduction in MTM compared to heterozygote wildtype negative control, an effect that localized to the mushroom body α′/β′ neurons (Figure 6A).

In order to determine the contribution of the “molecular memory switch” (Figure 1A) to aversive olfactory memory we expressed either the Ca^2+^-independent constitutively active form of *CaMKII-T287D* or Ca^2+^ dependent *CaMKII-T287A* (Park et al., 2002) in the mushroom body. Expression of *CaMKII-T287D* or *-T287A* either throughout the mushroom body (Figure 6B) or just in α′/β′ neurons caused a dramatic reduction in MTM (Figure 6C), with expression in the remaining α/β and γ neurons having no effect (Figure 6D), suggesting that the state of T287 autophosphorylation in α′/β′ is particularly important for memory formation. Restricted expression of *CaMKII-T287D* and *-T287A* transgenes to the adult mushroom body (Figure 6E) or just the adult α′/β′ (Figure 6F) but not the adult α/β and γ neurons (Figure 6G) was sufficient to cause the reduction in MTM. Negative control flies reared and tested at 18°C, displayed wildtype MTM (Figures 6H–J). In order to see if CaMKII inhibitory autophosphorylation is also important for memory formation (Figure 1B), we overexpressed a transgene with these phosphorylation sites (T306A T307A) blocked (Lu et al., 2003). *CaMKII-T306A T307A* overexpression throughout the mushroom body (Figure 6K) or just the α′/β′ neurons (Figure 6L) dramatically reduced MTM, while α/β and γ neuron expression had little effect (Figure 6M).

### CASK and CaMKII functionally interact to regulate middle-term memory formation

The main effect of CASK is to increase inhibitory phosphorylation of T306 T307 on endogenous CaMKII resulting in a decrease in endogenous kinase activity (Figure 1A; Lu et al., 2003; Hodge et al., 2006) and we show that mushroom body overexpression of *CASK* removes MTM. Conversely flies overexpressing the *uas-CaMKII-T306A T307A* transgene would have an opposing effect with inhibitory phosphorylation being blocked resulting in increased transgenic kinase activity (Figure 1B), again with mushroom body overexpression of *CaMKII-T306A T307A* removing MTM. As expression of the two transgenes are predicted to have opposite effects on CaMKII activity we decided to co-express *CASK* and *CaMKII-T306A T307A* to see if they counteract each other's effect and return memory to normal. Indeed flies expressing both transgenes in their mushroom body showed complete rescue of their memory deficit, confirming that CASK regulates CaMKII autophosphorylation during memory formation (Figure 6K). Expression of *CaMKII-T306A T307A* in just the α′/β′ neurons was not sufficient to rescue the *CASK* overexpression memory defect (Figure 6L). Expression of any combination of the transgenes in the α/β and γ neurons had no effect on MTM (Figure 6M). This data suggests that as for CASK, changes in CaMKII autophosphorylation are required throughout the adult mushroom body during memory formation and that the effect of CASK on MTM formation is through CASK's regulation of CaMKII autophosphorylation.

### CASK and CaMKII are required for long-term memory formation

**Figure 7:**
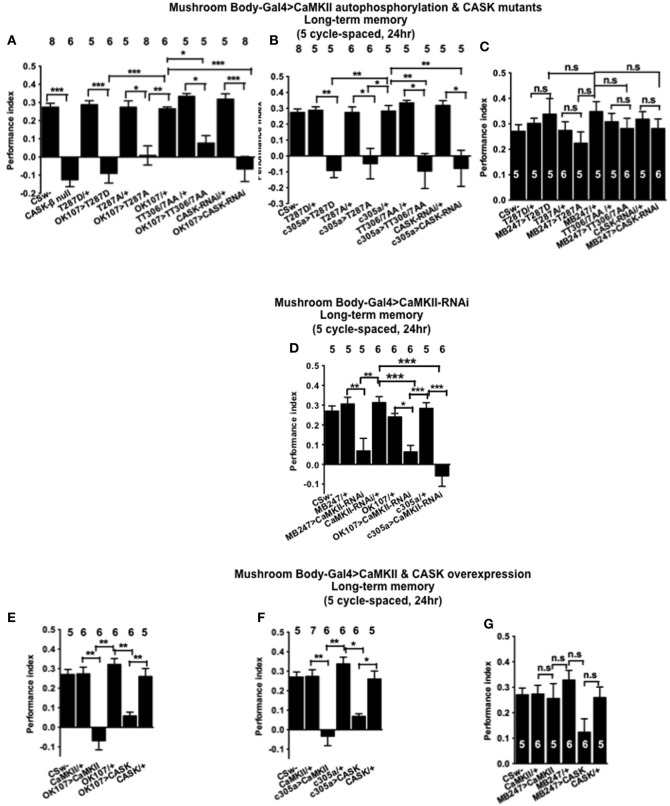
**CASK and CaMKII are required for long-term memory. (A)** Flies *null* for *CASK-β* or overexpressing *CASK-RNAi*, *CaMKII-T287D*, *CaMKII-T287A*, or *CaMKII-T306A T307A* throughout their mushroom body completely lacked LTM induced by five cycles of spaced training compared to heterozygous wildtype negative controls. **(B)** α′/β′ neuron *CASK* knockdown or overexpression of *CaMKII-T287D*, *CaMKII-T287A*, and *CaMKII-T306A T307A* was sufficient to cause this reduction in LTM. **(C)** Mushroom body α/β and γ neuron *CASK* knockdown or overexpression of *CaMKII-T287D*, *CaMKII-T287A*, and *CaMKII-T306A T307A* did not affect LTM. **(D)** Reduction of CaMKII throughout the mushroom body, in the α′/β′ or α/β and γ neurons decreased LTM. **(E)** Overexpression of CaMKII or CASK throughout the mushroom body decreased LTM. **(F)** Mushroom body α′/β′ overexpression of CaMKII or CASK decreased spaced LTM. **(G)** Overexpression of *CaMKII* or *CASK* in the mushroom body α/β and γ neurons (*MB247-Gal4*) did not affect LTM. Data were analyzed using One-Way ANOVA followed by a Tukey's *post-hoc* test. n.s. is not significant, (*p* > 0.05), ^*^p < 0.05, ^**^p < 0.01, and ^***^p < 0.001.

In order to determine the role of CASK and CaMKII autophosphorylation in LTM, flies were subjected to five cycles of spaced training which is known to produce a form of consolidated memory that is protein synthesis and cyclic-AMP response element binding protein (CREB) dependent (Tully et al., 1994). *CASK-β* null flies were not able to form LTM (Figure 7A). Similarly mushroom body *CASK* knockdown or overexpression of *CaMKII-T287D*, *T287A*, or *TT306/7AA* throughout the mushroom body (Figure 7A), just the α′/β′ (Figure 7B), but not the α/β and γ (Figure 7C) neurons reduced LTM compared to control. Previous studies have reported that *CaMKII* knockdown in α/β and γ mushroom body neurons or DAL neurons reduced LTM (Ashraf et al., 2006; Akalal et al., 2010; Chen et al., 2012). We therefore performed experiments using this *CaMKII-hairpin-RNAi* transgene but for the first time with a full complement of mushroom body neuron specific drivers (Figure 7D). Flies with a reduction of *CaMKII* in any of these sets of mushroom body neurons showed deficits in LTM indicating mushroom body CaMKII levels are crucial for normal LTM formation. This also demonstrates that the effect of changing the level of CaMKII as opposed to changing levels of autophosphorylated CaMKII can be qualitatively different in the α/β and γ neurons. Consistent with our MTM data we observed a similar reduction in LTM in flies overexpressing *CASK* or *CaMKII* throughout the mushroom body (Figure 7E), just in the α′/β′ neurons (Figure 7F), but not in the α/β and γ neurons (Figure 7G). Again this data is consistent with CASK function and CaMKII autophosphorylation in the α′/β′ neurons being critical for LTM memory formation.

### CASK and CaMKII levels and reduction of CaMKII autophosphorylation are required for anaesthesia resistant memory formation

**Figure 8:**
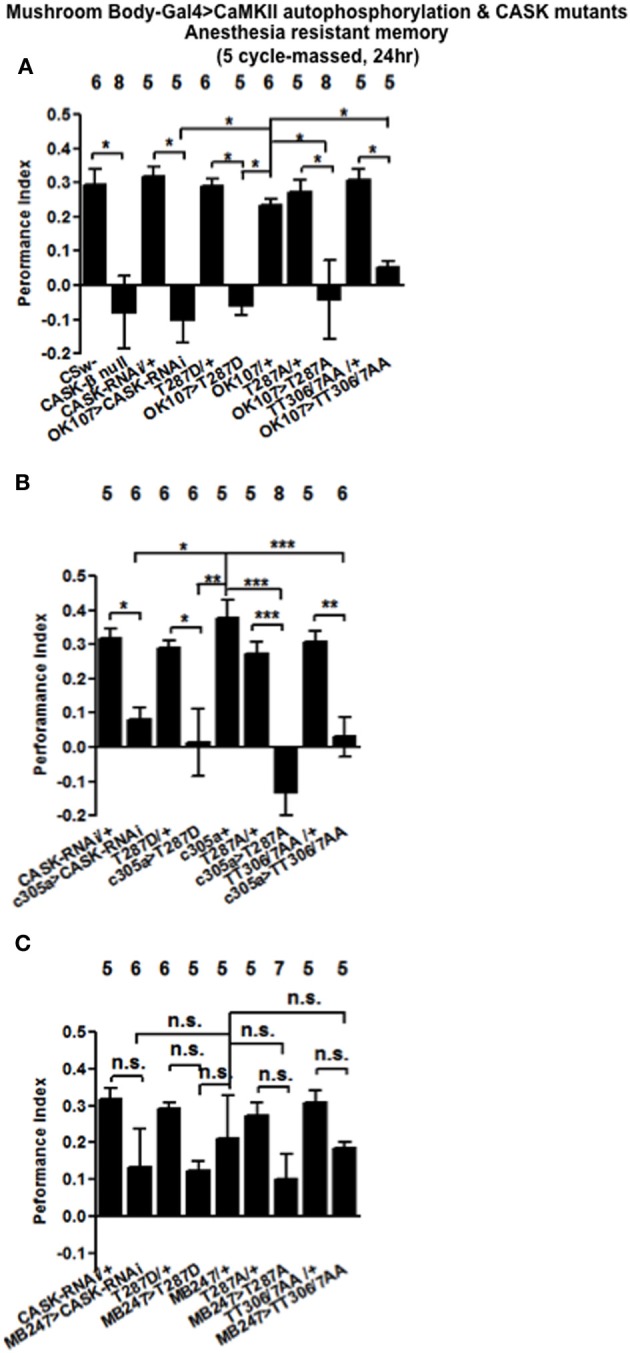
**CASK and CaMKII are required for anesthesia resistant memory. (A)** Flies null for *CASK-β* or expressing *CASK-RNAi*, *CaMKII-T287D*, or *CaMKII-T287A* throughout the mushroom body completely removed anesthesia resistant memory (ARM) tested 24 h after five cycles of massed training. **(B)** α′/β′ *CASK* knockdown or overexpression of *CaMKII-T287D*, *CaMKII-T287A*, or *CaMKII-T306A T307A* reduced ARM. **(C)** Mushroom body α/β and γ neuron *CASK* knockdown or overexpression of *CaMKII-T287D*, *CaMKII-T287A*, or *CaMKII-T306A T307A* did not affect ARM. Data were analyzed using One-Way ANOVA followed by a Tukey's *post-hoc* test. n.s. is not significant, (*p* > 0.05), ^*^p < 0.05, ^**^p < 0.01, and ^***^p < 0.001.

A second form of memory is generated by five cycles of training without rest intervals (massed training). This form of memory consists of anesthesia resistant memory (ARM) and is independent of CREB transcription (Tully et al., 1994). *CASK-β* nulls were not able to form ARM (Figure 8A), while mushroom body *CASK* knockdown or overexpression of *CaMKII-T287D*, *T287A*, or *TT306/7AA* throughout the mushroom body (Figure 8A), just the α′/β′ neurons (Figure 8B) but not the α/β and γ neurons (Figure 8C) neurons removed ARM. Our results are consistent with CASK function and CaMKII autophosphorylation in the α′/β′ neurons being critical for ARM formation.

### CASK and CaMKII regulate mushroom body neural activity

**Figure 9:**
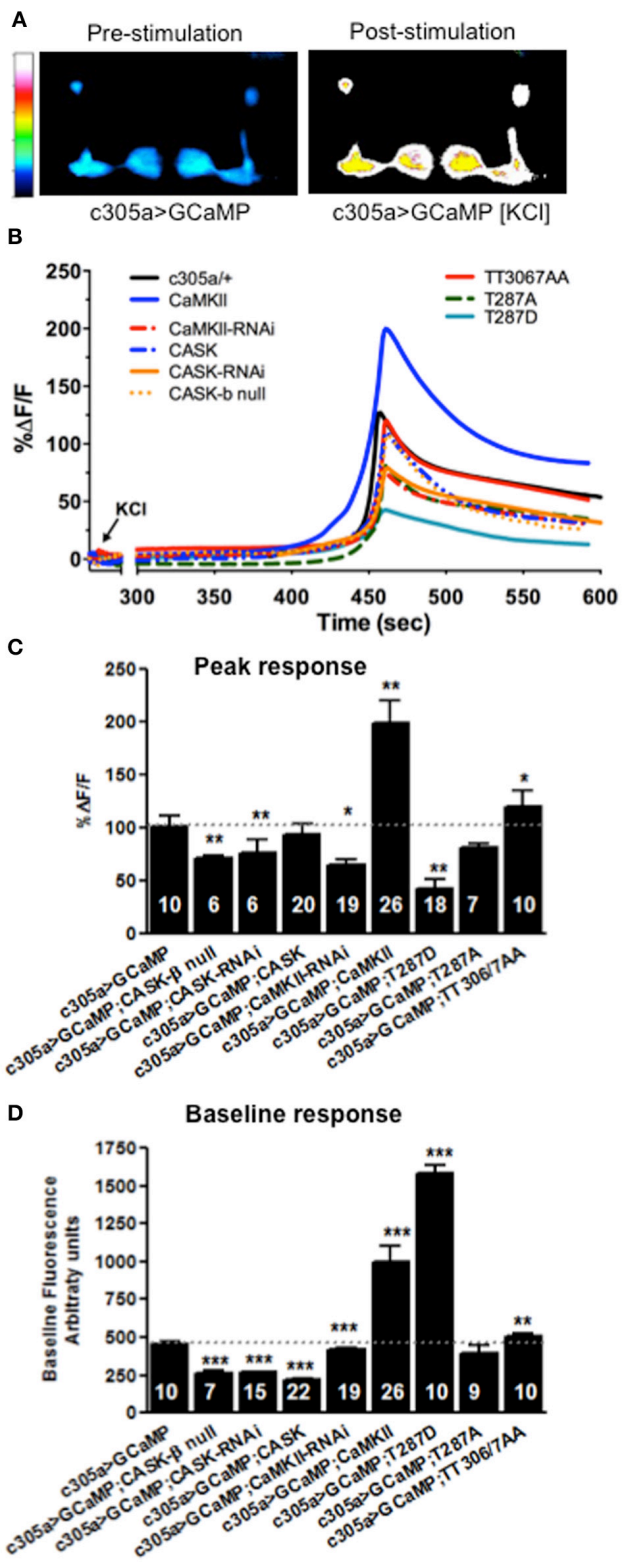
**CASK and CaMKII regulates dynamic changes in neural activity in mushroom body α′/β′ neurons. (A)** Color coded images of a fly brain showing GCaMP3.1 fluorescence in the mushroom body α′/β′ lobes using *c305a-Gal4* before and after application of depolarizing high [KCl]. **(B)** Traces showing averaged (*n* > 6) GCaMP3.1 fluorescence overtime in the α′/β′ mushroom body lobes (*c305a-Gal4*) co-expressing the different CASK and CaMKII transgenes or *CASK-β* null indicated compared to the negative control c305a/+ expressing GCaMP3 (solid black line). **(C)** Histogram showing that the % change in peak GCaMP3.1 fluorescence is reduced in *CASK-β* null and when *CASK-RNAi*, *CaMKII-RNAi*, or *CaMKII-T287D* were expressed in the α′/β′ neurons, while *CaMKII* overexpression increased the maximum response compared to negative control (*c305a-Gal4, uas-GCaMP3*) level (denoted by dotted line for comparison). **(D)** Histogram showing baseline Ca^2+^ levels were increased when *CaMKII, CaMKII-T287D*, or *CaMKII-T306A T307A* were overexpressed in α′/β′ neurons compared to negative control. *CASK-β* null or α′/β′ neuron overexpression of *CASK-RNAi*, *CASK*, or *CaMKII-RNAi* led to a reduction in baseline Ca^2+^ signaling. Data were analyzed using One-Way ANOVA followed by a Tukey's *post-hoc* test. n.s. is not significant, (*p* > 0.05), ^*^p < 0.05, ^**^p < 0.01, and ^***^p < 0.001.

Dynamic changes in neural activity and Ca^2+^ signaling in memory centers such as the mushroom body and hippocampus underlie memory formation (Lisman et al., 2002; Davis, 2011). Since CASK regulates MTM formation in adult mushroom body α′/β′ neurons (as labeled by *c305a-Gal4*), we set out to determine the physiological basis of this defect by measuring dynamic changes in Ca^2+^ signaling as reported by changes in fluorescence of the genetically encoded Ca^2+^ reporter, GCaMP3.1 in the relevant memory circuit (Tian et al., 2009). We imaged mushroom body Ca^2+^ induced fluorescence in response to acute application of high [K^+^] depolarizing solution that resulted in a robust increase in mushroom body intracellular Ca^2+^ levels (Figures 9A,B) and might reflect a proxy (although somewhat artificial) of the increase in synaptic activity occurring in α′/β′ neurons during memory formation in the behavioral experiments. *CASK-β* null or *CASK* knockdown in the α′/β′ neurons decreased maximum fluorescence (Figures 9B,C) indicating a disruption of neuronal signaling in the specific mushroom body neurons that cause the memory defect, consistent with this physiological change mediating the fly's inability to remember shown in Figures 3, 7, 8. *CaMKII* and *CaMKII-TT306/7AA* overexpression caused an increase in peak neural activity while reduced *CaMKII* caused a reduction in neural activity (Figures 9B,C), these bi-directional changes in neural activity provide an explanation for the disruption of memory seen with CaMKII misexpression in α′/β′ neurons (Figures 6–8). In addition overexpression of *CaMKII-T287D* also reduced the peak Ca^2+^ response in a similar manner to reduced *CASK*, consistent with reductions in CASK increasing levels of CaMKII autophosphorylated at T287 (Figure 1B; Hodge et al., 2006) and suggesting a physiological mechanism for memory deficit resulting from α′/β′ expression of *CaMKII-T287D* (Figures 6–8).

In order for relative changes in Ca^2+^ levels to encode information it would be expected that the baseline levels of Ca^2+^ would also be tightly regulated. Therefore, to see if plasticity molecules such as CaMKII and CASK are involved in setting basal Ca^2+^, GCaMP3 signals in α′/β′ neurons were measured under baseline conditions. Compared to wildtype (Figure 9D) overexpression of *CaMKII*, *CaMKII-T287D*, or *CaMKII-TT306/7AA* increased basal Ca^2+^ levels. Reduced *CaMKII* or *CASK* caused a decrease in basal Ca^2+^ levels α′/β′ neuron, while *CASK* overexpression also lowered baseline Ca^2+^ levels (Figure 9D), the later explaining the effect of α′/β′ overexpression on *CASK* on memory (Figures 3, 6, 7). Since overexpression of *CaMKII-T287D* already drives neurons into a very high Ca^2+^ state under basal conditions (Figure 9D), stimulation of the neurons may not be able to increase Ca^2+^ concentrations any further, reducing the change in Ca^2+^ concentration measured for peak response (Figure 9C). These results suggest CASK, CaMKII levels and autophosphorylation regulate basal and activity-dependent changes in Ca^2+^ signaling in the mushroom body α′/β′ neurons, revealing the likely neurophysiological basis for the disruption in memory found in these animals.

## Discussion

### CASK regulates CaMKII autophosphorylation in mushroom body α′/β′ neurons during middle-term memory formation

We found that *CASK-β* mutant flies that lack just the long isoform of CASK have reduced MTM, showing that the CaMK-like and L27 domains only present in this form of CASK (Figure 1C) are the key signaling domains required for regulating memory. Previous work has shown that CASK-β regulates CaMKII autophosphorylation by its CaMK-like domain (Figure 1A; Lu et al., 2003; Hodge et al., 2006), therefore based on this and the data presented here, it is likely that the way CASK functions in memory formation is via its control of CaMKII autophosphorylation mediated by its N-terminal CaMK-like domain. MTM formation was highly sensitive to the level and specific distribution of *CASK* in the mushroom body, with targeted reduction of *CASK* in the mushroom body α′/β′ neurons impairing memory, but with no apparent contribution from the α/β and γ neurons. Decreased levels of CASK are known to increase CaMKII-T287 autophosphorylation (Figure 1A; Lu et al., 2003; Hodge et al., 2006). Consistent with this, we found that direct overexpression of the *CaMKII-T287D* transgene in the α′/β′ neurons caused a similar reduction in MTM as knocking-down *CASK* in the same neurons. While expression of CaMKII-T287D in the α/β and γ neurons have no effect on MTM. Expression of CASK just in the α′/β′ neurons fully rescued the complete lack of memory in *CASK-β* null mutants to wildtype levels, showing CASK signaling in only the mushroom body α′/β′ neurons is necessary and sufficient for MTM formation.

We have also determined for the first time the effect of CaMKII overexpression on memory, showing α′/β′ neuron expression completely removed MTM. In addition reduction of CASK just in neurons that express CaMKII was sufficient to remove MTM. Furthermore, increasing CASK in α′/β′ neurons also greatly reduced MTM and decreased basal Ca^2+^ signaling. Such increases in CASK would be expected to block T287 autophosphorylation (Hodge et al., 2006), and indeed we found α′/β′ neuron T287A overexpression gave a similar MTM phenotype. The role of CASK and CaMKII-T287 autophosphorylation in the memory neurons is an acute physiological one as opposed to a developmental one, as reducing CASK or changing CaMKII-T287 autophosphorylation just in the adult mushroom body α′/β′ neurons was sufficient to remove memory. Recently a second pair of CaMKII autophosphorylation sites (TT306/7) has been shown to be important for the control of plasticity and memory in mammals (Figures 1A,B; Elgersma et al., 2002; Zhang et al., 2005). We found α′/β′ neuron *CaMKII-TT306/7AA* overexpression removed MTM. Lastly we demonstrated that overexpression of CASK completely rescued the memory deficit due to mushroom body overexpression of *CaMKII-T306/7AA* (Figure 6K). However, *CaMKII T306A T307A* expression in α′/β′ neurons was insufficient to rescue CASK overexpression (Figure 6L). The last result may suggest CASK does not regulate CaMKII T306 T307 in α′/β′ neurons, or perhaps the *c305a-Gal4* promoter may not have adequate strength or the exact spatiotemporal pattern required for both *CASK* and *CaMKII-T306A T307A* expression to make the fly remember as wildtype. Overall our data suggests that CASK regulates CaMKII autophosphorylation in a common pathway required for memory formation in the mushroom body.

### CASK regulates CaMKII autophosphorylation in the mushroom body α′/β#x02032; neurons during long-term memory formation

Previous work has shown mushroom body expression of CaMKII-T287D enhanced training but did not affect memory in the courtship conditioning assay, while CaMKII-T287A expression changed habituation and neuronal excitability, but resulted in no change in courtship conditioning memory (Mehren and Griffith, 2004). However, mushroom body expression of the *CaMKII-hairpin* transgene has been shown to decrease LTM using the olfactory aversive conditioning assay (Ashraf et al., 2006) and was associated with decreased mushroom body Ca^2+^ signaling (Akalal et al., 2010). The differences in effects of CaMKII on courtship and olfactory learning phenotypes maybe due to differences in the circuitry employed in the two memory tasks and also the timing of memory measured in the two assays. Recently CaMKII has been shown to undergo CREB-dependent gene transcription and translation in mushroom body and DAL neurons during LTM (Chen et al., 2012). Consistent with these studies we showed mushroom body expression of *CaMKII-hairpin* only affects LTM. In addition this is the only *CASK* or *CaMKII* transgene that gave a memory phenotype when expressed in the α/β or γ neuron, this suggests that LTM is particularly sensitive and requires a certain baseline level of CaMKII activity in every type of mushroom body neuron in order to form LTM. This is in contrast to transgenic manipulation of CaMKII autophosphorylation levels in the α/β or γ neuron that have no effect on LTM, possibly because the endogenous CaMKII in these neurons maybe adequate to support enough of the appropriate autophosphorylation activity to generate LTM. This is in contrast to the critical role of α′/β′ neurons that require the correct level of CASK, CaMKII, and CaMKII autophosphorylation in order to form LTM. Therefore, our data is consistent with the other studies showing α/β or γ (they did not test α′/β′) neuron expression of *CaMKII-RNAi* disrupts LTM, furthermore these studies showed that α/β or γ neuron *CaMKII-RNAi* expression decreased peak GCaMP3 Ca^2+^ response (Ashraf et al., 2006; Akalal et al., 2010).

We also measured a similar reduction in peak Ca^2+^ response in the in the α′/β′ neurons with *CaMKII-hairpin*; however, this was never tested for in the previous studies. We also found that the reciprocal CaMKII overexpression caused a large increase in peak Ca^2+^ response. Previous electrophysiological studies have shown neuronal expression of *CASK-RNAi* or *CaMKII-T287D* both decreased neural excitability in response to stimulation (Chen and Featherstone, 2011). Likewise we find expression of these transgenes caused a reduction in α′/β′ neuron peak Ca^2+^ signaling. Therefore, the GCaMP3 data is consistent with the current model of CASK regulation of CaMKII autophosphorylation (Figure 1A; Lu et al., 2003; Hodge et al., 2006).

Flies with the *CASK-β* null mutation or reduced CASK in the α′/β′ neurons reduced LTM. The LTM effects of CASK could be explained by its role in transcriptional activation of various plasticity molecules including NMDA receptors (Wang et al., 2002; Huang and Hsueh, 2009). NMDA receptors have recently been shown to be required for LTM in *Drosophila* (Wu et al., 2007). Furthermore, CaMKII itself is known to be a direct target of NMDA receptor activation (Thalhammer et al., 2006) leading to increased CaMKII-T286 autophosphorylation and subsequent phosphorylation and activation of molecules required for synaptic plasticity and LTM (Trinidad et al., 2006). At present there is no evidence that *Drosophila* CASK translocates to the nucleus; however, the effects of CASK on LTM maybe through changes in *CaMKII* expression that is known to occur during LTM (Ashraf et al., 2006; Akalal et al., 2010). We show that the CaMKII molecular memory switch (pT287) is required for mushroom body LTM formation with phospho-mimic or block removing both ARM and LTM. Again this seems to be an evolutionarily conserved memory mechanism with T286 mutant mice also not being able to form LTM after massed training (Irvine et al., 2011).

### Human CASK function in mushroom body α′/β′ neurons restores memory performance of *CASK* null flies

Point mutations in human CASK have been associated with neurological and cognitive defects, including severe learning difficulties resulting from mutations in the CaMK-like and SH3 domains (Najm et al., 2008; Piluso et al., 2009; Tarpey et al., 2009). Recently CASK mutation has been shown to cause a number of cognitive defects in flies including disrupted sleep and place preference (Slawson et al., 2011; Donelson et al., 2012). In addition to these defects we show that CASK mutants with deletion of the CaMK-like and L27 domains have extreme impairment of MTM and LTM formation. Furthermore, we show that α′/β′ neuron overexpression of human CASK can fully substitute for the lack of *Drosophila* CASK-β and rescue the *CASK-β* mutant memory defect to wildtype. This demonstrates that neuronal function of CASK is conserved between *Drosophila* to human, validating the use of this model to understand CASK function in both the healthy and diseased brain.

In conclusion we have demonstrated that CASK functions in the α′/β′ neurons required for memory formation and levels of CaMKII autophosphorylation are critical for MTM and LTM. We show bi-directional changes in CaMKII and CASK levels in α′/β′ neurons result in disrupted Ca^2+^ signaling dynamics. Our results show that CASK regulates CaMKII autophosphorylation in memory formation.

### Conflict of interest statement

The authors declare that the research was conducted in the absence of any commercial or financial relationships that could be construed as a potential conflict of interest.
